# Prognostic Assessment in High-Grade Soft-Tissue Sarcoma Patients: A Comparison of Semantic Image Analysis and Radiomics

**DOI:** 10.3390/cancers13081929

**Published:** 2021-04-16

**Authors:** Jan C. Peeken, Jan Neumann, Rebecca Asadpour, Yannik Leonhardt, Joao R. Moreira, Daniel S. Hippe, Olena Klymenko, Sarah C. Foreman, Claudio E. von Schacky, Matthew B. Spraker, Stephanie K. Schaub, Hendrik Dapper, Carolin Knebel, Nina A. Mayr, Henry C. Woodruff, Philippe Lambin, Matthew J. Nyflot, Alexandra S. Gersing, Stephanie E. Combs

**Affiliations:** 1 Department of Radiation Oncology, Klinikum rechts der Isar, Technical University of Munich (TUM), Ismaninger Straße 22, 81675 Munich, Germany; rebecca.asadpour@tum.de (R.A.); olena.klymenko@mri.tum.de (O.K.); hendrik.dapper@mri.tum.de (H.D.); stephanie.combs@tum.de (S.E.C.); 2 Institute of Radiation Medicine (IRM), Department of Radiation Sciences (DRS), Helmholtz Zentrum München, 85764 München, Germany; 3 Deutsches Konsortium für Translationale Krebsforschung (DKTK), Partner Site Munich, Germany; 4 Department of Precision Medicine, GROW—School for Oncology and Developmental Biology, Maastricht University, 6200 MD Maastricht, The Netherlands; h.woodruff@maastrichtuniversity.nl (H.C.W.); philippe.lambin@maastrichtuniversity.nl (P.L.); 5 Department of Radiology, Klinikum rechts der Isar, Technical University of Munich (TUM), 81675 Munich, Germany; jan.neumann@tum.de (J.N.); yannik.leonhardt@tum.de (Y.L.); ricardounifesp73@gmail.com (J.R.M.); sarah.foreman@tum.de (S.C.F.); c.schacky@tum.de (C.E.v.S.); alexandra.gersing@tum.de (A.S.G.); 6 Department of Radiation Oncology, University of Washington, Seattle, WA 98195, USA; dhippe@uw.edu (D.S.H.); skschaub@uw.edu (S.K.S.); ninamayr@uw.edu (N.A.M.); nyflot@uw.edu (M.J.N.); 7 Department of Radiation Oncology, Washington University in St. Louis, St. Louis, MO 63110, USA; mspraker@wustl.edu; 8 Department of Orthopedics and Sports Orthopedics, Klinikum rechts der Isar, Technical University of Munich (TUM), 81675 Munich, Germany; carolin.knebel@mri.tum.de; 9 Department of Radiology and Nuclear Imaging, GROW—School for Oncology and Developmental Biology, Maastricht University Medical Centre, 6229 HX Maastricht, The Netherlands; 10 Department of Radiology, University of Washington, Seattle, WA 98195, USA

**Keywords:** radiomics, machine learning, soft-tissue sarcomas, radiology, MRI, tail sign, prognosis, elastic net regression

## Abstract

**Simple Summary:**

Soft-tissue sarcomas constitute a rare cancer type, with approximately 40% of patients experiencing disease recurrence. There is a need for a better identification of patients with especially aggressive tumors. Previous research demonstrated that the qualitative assessment of imaging data by radiologists (“semantic features”) and the algorithm-based analysis of imaging data (termed “radiomics”) may help to achieve a more thorough identification of patients at high risk for cancer-specific mortality. In this work, we compared the performance of predictions of patients’ survival based on semantic features extracted by radiologists with a “radiomic” approach. While some semantic features were helpful to identify high-risk patients, the radiomic approach achieved an overall improved ability to identify patients at high risk. For the radiomic prediction, only one MRI sequence was sufficient and an MRI sequence without the need for contrast agent achieved good predictive performance.

**Abstract:**

Background: In patients with soft-tissue sarcomas of the extremities, the treatment decision is currently regularly based on tumor grading and size. The imaging-based analysis may pose an alternative way to stratify patients’ risk. In this work, we compared the value of MRI-based radiomics with expert-derived semantic imaging features for the prediction of overall survival (OS). Methods: Fat-saturated T2-weighted sequences (T2FS) and contrast-enhanced T1-weighted fat-saturated (T1FSGd) sequences were collected from two independent retrospective cohorts (training: 108 patients; testing: 71 patients). After preprocessing, 105 radiomic features were extracted. Semantic imaging features were determined by three independent radiologists. Three machine learning techniques (elastic net regression (ENR), least absolute shrinkage and selection operator, and random survival forest) were compared to predict OS. Results: ENR models achieved the best predictive performance. Histologies and clinical staging differed significantly between both cohorts. The semantic prognostic model achieved a predictive performance with a C-index of 0.58 within the test set. This was worse compared to a clinical staging system (C-index: 0.61) and the radiomic models (C-indices: T1FSGd: 0.64, T2FS: 0.63). Both radiomic models achieved significant patient stratification. Conclusions: T2FS and T1FSGd-based radiomic models outperformed semantic imaging features for prognostic assessment.

## 1. Introduction

Soft-tissue sarcomas (STS) constitute a rare malignant entity comprising 1% of all cancers [1]. Patient outcome and therapeutic management differ significantly between anatomic sites of the primary tumor (1). In patients with high-risk STS of the extremities, resection is commonly combined with neoadjuvant or adjuvant radiotherapy (RT) for improved local progression-free survival (LPFS) and overall survival (OS) [2,3]. In contrast to the high LPFS rates of up to 94%, current therapy regimens achieve comparably low OS, with low distant progression-free survival (DPFS) rates [4,5,6,7].

There are large research efforts underway to find biomarkers for the prediction of therapy response, disease progression, and survival. Semantic imaging features have been shown to correlate with prognosis in multiple cancer entities [8,9,10]. In STS patients with diverse histologies, Crombé et al. demonstrated significant correlations of three semantic features (peritumoral enhancement, necrosis, heterogenous Tw2 signal intensity) with high tumor grading, OS, and DPFS [11].

The relatively novel field of imaging-based “radiomics” constitutes an alternative approach to characterize tissues, with the advantage of analyzing the whole tumor volume instead of only a focal biopsy sample. It is defined as an algorithm-based large-scale quantitative analysis of imaging features [12,13,14,15]. Such imaging biomarkers were shown to predict survival, tumor progression, spatial infiltration, and molecular aberrations in a multitude of cancer types [16,17,18,19,20,21]. In a recent publication, Spraker et al. did demonstrate a prognostic potential of contrast-enhanced and fat-saturated T1-weighted (T1FSGd) sequence-based radiomics in STS patients [22]. An earlier pilot study showed a predictive capability for distant metastases by applying radiomics to T1-weighted and T2-weighted fat-saturated (T2FS) sequences that were fused with ^18^F-fluorodeoxyglucose positron emission tomography data [23].

The scope of this study was to compare the benefit of expert-derived semantic imaging features with radiomic models based on multiparametric MRI-scans, combining T1FSGd and T2FS sequences. The predictive value for OS was assessed and compared to clinical baseline models. The resulting models were validated in an external patient cohort. Finally, the importance of single semantic features was assessed.

## 2. Materials and Methods

### 2.1. Patients

Two independent patient cohorts from the University of Washington, Seattle, WA, USA (UW) and the Technical University of Munich, Munich, Germany (TUM) were used for radiomic model training and testing, respectively. Patient records of patients with STS of the extremities or trunk were analyzed for patients’ age, grading, and TNM-staging. All patients received either preoperative, postoperative, or definitive RT, curative in intent, with or without chemotherapy. Exclusion criteria were low-grade, incomplete imaging data; previous RT; primary bone sarcomas; Ewing sarcomas; rhabdomyosarcomas; distant metastases at diagnosis (M1); and endoprosthesis-dependent MRI artifacts. See Figure S1 for a patient workflow. If an exclusion-relevant criterium was missing, the patient was excluded. In the final patient cohort, no modeling-specific data were missing. OS was calculated from the initial pathologic diagnosis to the time point of death or the time point of censoring. Data reporting follows the TRIPOD recommendations (Table S10: TRIPOD checklist) [24].

### 2.2. Image Acquisition and Definition of Volume of Interests

**Figure 1:**
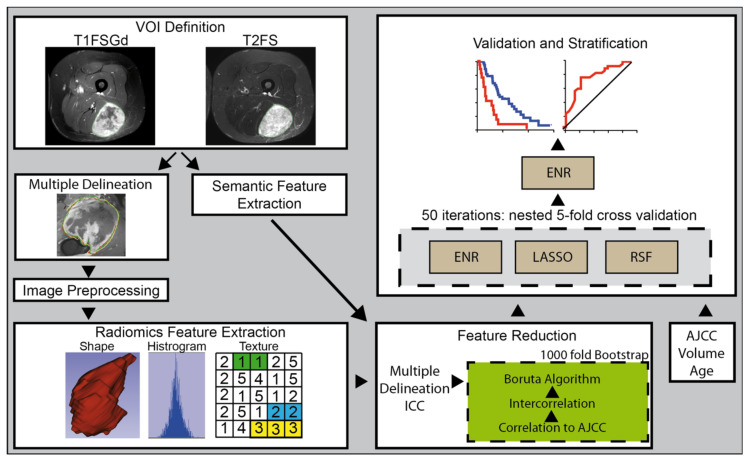
Radiomics Workflow. Abbreviations: AJCC: American Joint Committee on Cancer and the International Union for Cancer Control (8th edition), DCA: decision curve analysis, ENR: elastic net regression, ICC: intraclass coefficient, LASSO: least absolute shrinkage and selection operator, T1FSGd: T1-weighted fat-saturated with gadolinium, T2FS: T2-weighted fat-saturated, VOI: volume of interest.

Each included patient received pre-RT MRI scans. See Table S2 for acquisition parameters and scan planes. Tumor segmentation was performed using MIM software version 6.6 (MIM Software Inc, Cleveland, USA), Eclipse 13.0 (Varian Medical Systems, Palo Alto, USA), iplan RT 4.1.2 (Brainlab, Munich, Germany), and 3D Slicer (3D Slicer, Version 4.8 stable release). All segmentations were transformed to masks. The primary tumor as the volume of interest (VOI) was manually segmented by JCP, by adapting existing expert segmentations from RT treatment planning in the TUM cohort. In the UW cohort, segmentation was performed by MBS, MM, JCP, and TC. Edematous changes were not included in the VOI. To compensate for operator-dependent bias, multiple delineations were performed for 21 randomly selected patients by three radiation oncologists (RA, MBS, JCP) in the UW cohort (see Figure 1). The DiceComputation module of 3D Slicer was used to calculate the Dice coefficient (DC) [25].

### 2.3. Image Preprocessing and Radiomic Feature Extraction

N4ITK MRI bias field correction was applied to each imaging study using the Slicer3D implementation to compensate for non-uniform intensity caused by field inhomogeneity [26]. The pyradiomics package (Version 2.2) implemented in Python (3.7) was used for all preprocessing steps and radiomic feature extractions [27]. All radiomic features were calculated consistent with the Imaging Biomarker Standardization Initiative (IBSI) [28]. Preprocessing was conducted before image analysis. Due to the relative nature of MRI intensity values, image discretization was performed with a fixed bin width of 10. Intensity normalization was performed by redistributing the image at the mean with the standard deviation and a scale of 100. Isotropic resampling to a voxel size of 1x1x1 mm was performed by using Bspline interpolation. No voxel array shift was performed to be consistent with the IBSI guidelines. As current data point towards impaired reproducibility of filter-based features, we extracted features only from the original version of the image [29]. In sum, 105 features were extracted from the original image of each sequence within the segmented label map, including first-order features, shape features, and texture features. Texture features included “Gray Level Co-occurrence Matrix” (GLCM) features, “gray level size zone matrix” (GLSZM) features, “gray level run length matrix” (GLRLM) features, “neighboring gray tone difference matrix” (NGTDM) features, and “gray level dependence matrix” (GLDM) features, leading to a total feature number of 210. A detailed listing of extracted features is shown in Table S3.

### 2.4. ComBat Batch Harmonization

ComBatHarmonization has been proposed as a method for the correction of batch effects among multicenter radiomic cohorts [30,31]. Its value to improve reproducibility between different centers has been shown in multiple studies [32,33,34]. The additive and multiplicative batch effects on a given feature distribution are estimated using a maximum likelihood approach. We applied nonparametric ComBatHarmonization (https://github.com/Jfortin1/ComBatHarmonization, accessed on 16 April 2020), correcting for MRI scanner models with mean site effects adjustment. We compensated for the MRI scanner type due to the small patient number.

### 2.5. Semantic Imaging Features

The MR imaging examinations were independently assessed by three radiologists (8 years, 7 years, and 3 years of experience in musculoskeletal radiology, respectively). The radiologists were blinded to the clinical information as well as the histological diagnosis. Two of the radiologists exclusively read imaging studies of one of the cohorts. The third radiologist, however, read image studies from both cohorts. Ten patients within the TUM cohorts were read by all three radiologists to assess the interrater agreement. The following radiological features were assessed in the study (see Table 1 for a description of all features) [35,36,37,38]: anatomical region of tumor (chest/back, leg, foot, arm, hand, gluteal/pelvic region), localization (epifascial, subfascial, or epi- and subfascial; intramuscular; intermuscular or inter-/intramuscular), tumor morphology (multinodular (more than one separate mass in the same region), mass-like (round or oval mass) or with superficial expansion along membranes/surfaces)), and tumor margins (well-defined, locally infiltrating or diffusely infiltrating). Moreover, on T1-weighted images with fat saturation and contrast enhancement, volume of contrast-enhancing tumor tissue (extent of enhancement < 1/3, 1/3–2/3, or > 2/3 of tumor volume), enhancement pattern (homogeneous/inhomogeneous), presence of vascularization (present/absent), necrosis (present/absent), perilesional contrast enhancement (present/absent), and the tail sign (defined as a well-defined, pointed curvilinear formation at least 10 mm in length on T1FSGd images) were assessed. The maximal tumor diameter without tail sign (in mm) was measured on the T1FSGd images with contrast enhancement. On the T2FS images, presence of perilesional edema (present/absent), diameter of edema (in mm), extent of edema (diffuse or circumscribed), dominant T2FS signal intensity (hypointense/isointense/hyperintense), and dominant T2FS signal pattern (homogeneous or inhomogeneous) were graded. Before modeling features were one-hot encoded to dummy variables.

### 2.6. Modeling Strategy

Three common machine learning techniques established for survival analysis were trained and compared to predict OS: random survival forest (RSF), least absolute shrinkage and selection operator (LASSO), and elastic net regression (ENR) [39,40,41]. As a first feature reduction step, all features susceptible to variations in the subset of patients that received three independent segmentations were excluded. As a threshold, an intraclass correlation coefficient (ICC) (3,1) of 0.8 was used. The remaining features (T1FSGd: 103, T2FS: 72) were used as input for the modeling pipeline. All three models were developed within the same pipeline. The pipeline combined (1.) additional feature reduction and (2.) model training (see Figure 1). (1.) The following feature reduction procedure was performed using 1000 bootstrap samples. Features correlated to the clinical American Joint Committee on Cancer and the International Union for Cancer Control (AJCC) (8th edition) staging groups defined by a Spearman correlation coefficient of greater than 0.8 were excluded [42]. Secondly, highly intercorrelated features defined by a Spearman correlation coefficient of greater than 0.8 were excluded. For the identified highly correlated feature pairs, the feature with the highest mean correlation to all remaining features was excluded. Thirdly, the Boruta algorithm was applied to filter the most relevant features [43,44]. The features were ranked according to the frequency of their selection in the 1000 bootstrap runs. The final feature set was defined as the top-ranking features. The final feature number per model was defined as the median feature number selected over all bootstrap runs.

To compare the performance of the three machine learning models, 50 iterations of 5-fold nested cross-validation was performed using the UW cohort (referred to as “training cohort”). All three models were developed using the mlr3 package [45]. Hyperparameters were optimized using random search and 25 evaluations. The RSF was developed with 1000 trees. Hyperparameter optimization was conducted for node size (search space 3–20) and the number of input variables randomly chosen at each node (mtry) (search space 2–10). For ENR, alpha (search space 0.05–1.0) and lambda were optimized. For LASSO, alpha was set to 1 and lambda was optimized. No correction for unbalanced data was applied. After comparison of the modeling strategies (see 3.2) a final set of ENR models was retrained on the training cohort using 5-fold cross-validation and tested on the TUM cohort (referred to as “testing cohort”).

In total, three different radiomic models were developed: *Radiomics-T1* based on T1FSGd-derived radiomic features, *Radiomics-T2* based on T2FS-derived radiomic features, and *Radiomics-T1T2* combining both feature sets. A semantic model (*Semantic*) was trained using the semantic features as input. Finally, a model combing semantic and radiomic features (*Radiomics-T1T2+Semantic*) was developed. Combined models were trained and tested as multivariate cox regression models using AJCC-stage, age, and the predictors of the developed models as an input. The concordance index (C-index) was calculated to assess model performance. The 95% confidence interval was estimated using 1000-fold bootstrapping.

To assess the influence of independent patient cohorts on model performance, we recalculated the final models on a new training set, mixing randomly chosen patients from both institutions with equal size and event numbers compared to the original training cohort. The remaining mixed patients were used as a test cohort.

### 2.7. Statistical Analysis

Statistical analysis and modeling were performed using R (version 3.4.0, R core team, Vienna, Austria). See Table S4 for R packages and versions. Fleiss Kappa and ICC were calculated to test for interrater agreement. Kaplan–Meier survival curves were generated to analyze stratified patient subgroups. The cutoff to split patients into low-risk and high-risk patients was defined as the median of the predictors in the training set. Statistical significance was tested using the Log-rank test. Time-dependent area under the receiver operating characteristic (ROC) curve (AUC) and calibration curves were plotted to characterize model performances. Bonferroni correction was performed in cases of multiple testing as specified. A *p*-value below 0.05 was regarded as significant.

## 3. Results

### 3.1. Patient Characteristics, Histology and VOI Definition

Overall, patient demographics were similar between both cohorts (Table 2); however, STS histologies were different between the cohorts (*p* < 0.001) (Table S1). Pleomorphic sarcoma was the most frequent histology in both groups, although with a larger proportion in the training set (training: 45%, testing: 34%). The second and third most frequent histologies were leiomyosarcoma (11%) and spindle cell carcinoma (10%) in the training cohort and myxofibrosarcoma (18%) and synovial sarcoma (13%) in the testing cohort. There were more unfavorable characteristics in the testing cohort with a larger proportion of AJCC stage 3 patients and 5 patients (7%) treated in a recurrent setting. Significantly more patients from the training cohort received chemotherapy. In the testing cohort, the delivered total RT dose was significantly higher than in the training cohort and a higher number of patients received definitive RT (6% vs 1%). There was an overall high similarity between multiple tumor target volume delineations performed by the three independent operators with a mean Dice similarity coefficient (DSC) of 0.92 (range (min–max): 0.81–0.96).

### 3.2. Interrater Agreement of Semantic Imaging Features

Ten randomly chosen patients were read by the three independent radiologists. Nominal and ordinal features achieved a median Fleiss Kappa of 0.524 (range: 0.035–1.00). Overall, five features achieved a “substantial/good” agreement (Kappa > 0.60), five features achieved a “moderate” agreement (0.40–0.60), and three features achieved only “slight/fair” agreements (0.00–0.40), as defined by Altmann and Landis [46,47]. The two continuous measures were correlated with a median ICC of 0.833 (range: 0.138–0.846). Table S5 displays all Kappa and ICC values for each feature.

### 3.3. Comparison of Semantic Imaging Features and Radiomics for Prediction of Overall Survival

The feature reduction pipeline yielded a median number of 12 (*Radiomics-T1*, range: 4–23), 10 (*Radiomics-T2*, range: 3–16), 11 (*Radiomics-T1T2*, range: 7–27), and 9 (*Semantic*, range: 3–16) features that were used for the prediction models. *Radiomics-T1T2+Semantic* combined features of *Radiomics-T1T2* and *Semantic*. The selected features are listed in Table S7.

**Figure 2:**
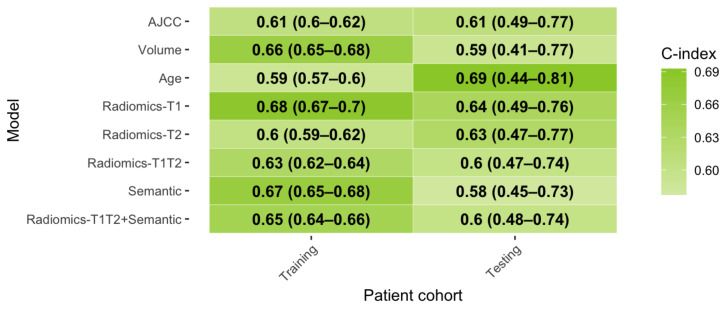
Prognostic performance of developed ENR models. **Abbreviations**: AJCC: American Joint Committee on Cancer and the International Union for Cancer Control (8th edition), C-index: concordance-index, OS: overall survival.

Three ML modeling strategies were applied and compared to predict OS. The ML techniques were ranked in order of the predictive performance in the external cross-validation folds for each model. ENR, RSF, and LASSO achieved a mean rank of 1.4, 2.2, and 2.4, respectively. Table S6 lists the C-indices per ML technique and feature set. Due to the overall better outcome of the ENR model, it was applied for further analyses and validated on the independent test set. See Figure 2 for the respective C-index values.

During nested cross-validation within the training set, *Radiomics-T1* achieved a superior performance (C-index: 0.68) compared to *Radiomics-T2* (C-index: 0.60). In comparison, the *Semantic* model achieved a performance comparable to *Radiomics-T1* in the training set (C-index: 0.67).

In the external test set, however, both radiomic models performed similarly (*Radiomics-T1*: C-index: 0.64, *Radiomics-T2*: 0.63). Combining both feature sets (*Radiomics-T1T2*) did not trigger an improved testing performance (C-index: 0.60). The *Semantic* model failed to reproduce the predictive performance from the training set (C-index: 0.58). A model combining all imaging features *Radiomics-T1T2+Semantic* did not improve performance further (C-index:0.6). For comparison, three clinical baseline models were computed. The AJCC staging system (C-index: 0.61), and tumor volume (C-index: 0.59) showed worse performance compared to the radiomic models in the test set. Age achieved the highest C-index (0.69) among all predictors in the test set. Figures S2 and S3 depict the time-dependent AUC and calibration curves, respectively.

**Figure 3:**
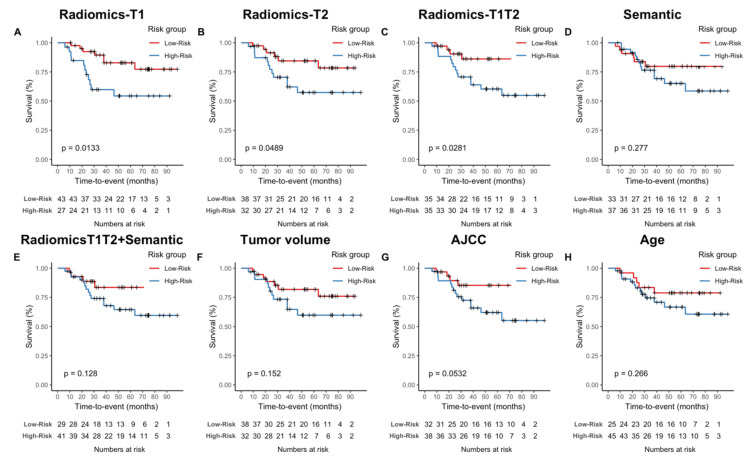
Kaplan–Meier survival analyses of developed models in the testing cohort. (**A**–**F**) Kaplan–Meier survival curves. Cohorts were split based on the median predictor value determined on the training cohort. (**G**,**H**) As a consequence, the AJCC staging system was split between stage III and stages IIA/B.

The propensity to achieve patient stratification was evaluated using Kaplan—Meier analysis (Figure 3). In the testing cohort, all three radiomic models achieved a significant separation of survival curves (curves were split at the median of the training cohort predictors). For *Semantic*, *Radiomics-T1T2+Semantic*, *Volume*, and *Age*, there was no significant risk stratification. For *AJCC*, a trend towards significance could be observed (*p* = 0.0532).

### 3.4. Relevance of Combined Clinical-Imaging Models

**Figure 4:**
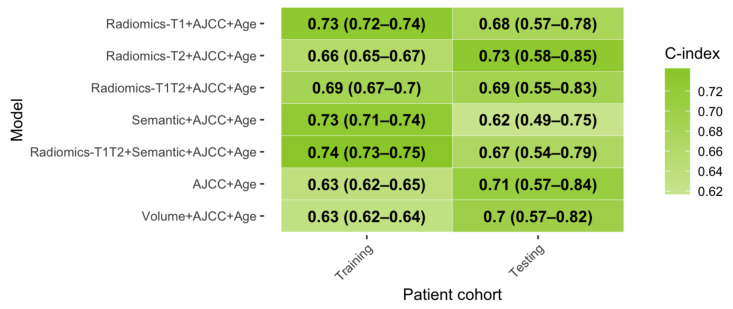
Prognostic performance of combined models. Abbreviations: AJCC: American Joint Committee on Cancer and the International Union for Cancer Control (8th edition), C-index: concordance-index, OS: overall survival.

**Figure 5:**
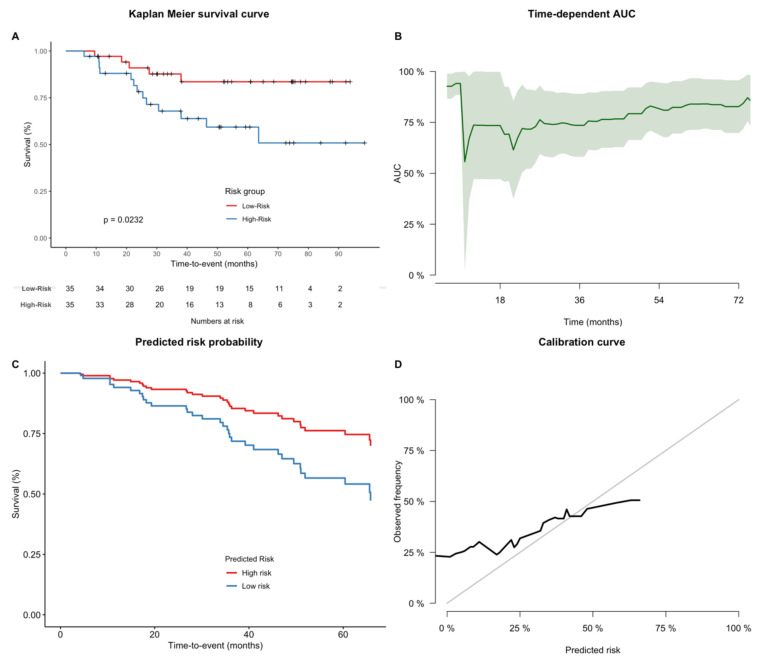
Prognostic performance of the *Radiomics-T2+AJCC+Age* model in the testing cohort. (**A**) Kaplan–Meier survival curve. Cohorts were split based on the median predictor value determined on the training cohort. (**B**) Time-dependent area under the receiver operating curve (AUC). (**C**) Predicted survival probabilities of the Cox proportional hazards model. (**D**) Calibration curve of the Cox proportional hazards model.

To test for a potential incremental benefit, the radiomic and semantic models were combined with the AJCC staging system and patients’ age (Figure 4). *AJCC+Age* alone achieved the best performance in the test set so far (C-index: 0.71). Adding the *Radiomics-T2* model improved the predictive performance further by +0.02 (*Radiomics-T2+AJCC+Age*: C-index: 0.73). Models combining AJCC and age with the *Semantic* model (C-index: 0.62) or the *Radiomics-T1* model failed to increase performance (C-index: 0.67). The combined *Radiomics-T2+AJCC+Age* model achieved significant patient stratification in Kaplan–Meier analysis (Figure 5). The mean time-dependent AUC was 0.79.

### 3.5. Relevance of Single Imaging Parameters

To investigate the prognostic value of isolated semantic features, univariate Cox proportional hazards regression was performed on the combined cohort (Table 3). Three features, including “maximal diameter without tail” (*p* = 0.022), “necrosis” (*p* = 0.039), and “edema perilesional” (*p* = 0.043), were significantly associated with OS (without correction for multiple testing). When testing for an interaction between patient cohorts, none of the interactions reached statistical significance.

**Figure 6:**
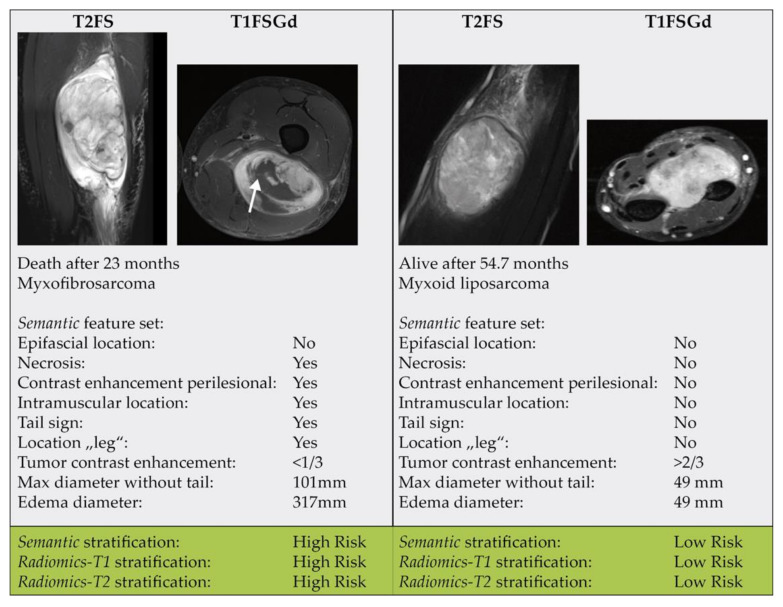
Two exemplary patient cases.

All semantic parameters that were found to be significant in the combined cohort were also included in the final feature reduction set. Besides, the parameters “epifascial and intramuscular location”, “contrast enhancement perilesional”, as well as the anatomic location “leg” were selected. See Table S7 for the coefficients of the final models. Figure 6 shows two exemplary cases.

In the *Radiomics-T1* model, only the features “Firstorder-Mean” and “Shape-SurfaceArea” retained non-zero coefficients. The *Radiomics-T2* model included several GLSZM and GLDM based features with non-zero coefficients.

### 3.6. Analysis of Model Calibration

Besides the C-index, we analyzed calibration curves of the developed models. Table S8 depicts Brier scores of all models. *AJCC* and *Age* had the lowest Brier scores (24 and 30, respectively). *AJCC* showed the most monotonous slope. Radiomic models showed Brier scores ranging from 88 to 114, while models comprising semantic features had the worst calibrations (Brier scores from 370–702). For combined models, however, the predicted risk showed a better correlation with observed frequency with lower Brier scores (Brier scores from 37 to 56) (Figure 5D, Figure S4). For all models, larger predicted risks were not well correlated to high observed frequencies.

### 3.7. Assessment of the Impact of the Independence of the Test Cohort

Retraining and testing of prediction models on randomly selected training and testing cohorts combining patients form both institutions led to a better reproducibility of the developed models. Both radiomic models *Radiomics-T1* and *Radiomics-T2* achieved a testing AUC of 0.63, which was equal to the training performance. Interestingly, the *Semantic* model also achieved a higher reproducibility, with a testing AUC of 0.63 and a difference of –0.01 compared to the training set.

## 4. Discussion

In this work, we demonstrated that a standardized semantic prognostic model predicted survival with moderate performance. Radiomic models achieved an added benefit in predicting OS relative to semantic features. Interestingly, the performance of *Radiomics-T1* and *Radiomics-T2* was comparable in terms of C-index. Both models achieved significant risk stratifications in the testing cohort. Importantly, combining the T1FSGd and T2FS radiomic feature sets with or without the semantic features did not trigger an additional benefit. The best combined model using T2FS-based radiomics features (*Radiomics-T2+AJCC+Age*) achieved a modest incremental benefit above the clinical model (*AJCC+Age*) alone.

Multiple studies previously demonstrated significant associations of semantic imaging features with prognosis in STS patients. For instance, in a previous study, we demonstrated that semantic features, such as tumor size, septa thickness, contrast enhancement could distinguish atypical lipomatous tumors from benign lipomas [38]. The presence of perilesional edema and T2 heterogeneity of liposarcomas detected with MRI predicted pulmonary metastases in a previous study [48]. Moreover, perilesional edema detected on MR images of myxofibrosarcomas correlated significantly with a poor OS rate [49]. The “tail sign” describing tumor cell infiltrations extending from the primary tumor along the deep fascia has been shown to correlate with LPFS in myxofibrosarcoma and undifferentiated sarcomas [37]. In a non-histology-specific STS cohort, the three semantic features found by Crombé et al. (peritumoral enhancement, necrosis, heterogenous T2-weighted signal intensity) were also correlated to OS and DPFS [11]. In our analysis, necrosis was associated with worse survival as well. Besides necrosis, peritumoral enhancement and the tail sign were selected into the final *Semantic* feature set, signaling a predictive relevance in our study, too.

In the univariate analysis, we could identify significant prognostic semantic features. However, several factors may have contributed to negatively influencing the predictive performance of the developed combined semantic prediction model. First, a substantial proportion of the semantic features achieved only moderate interrater agreement and may have hindered effective reproduction in the testing cohort. Second, semantic feature assessment may have been impaired by image acquisition and reconstruction parameters as it is known for radiomic features. ComBatHarmonization has been proposed to reduce the variability of radiomic features [33]. Potential novel standardization techniques may help to harmonize between readers and/or imaging acquisition characteristics. Third, with the availability of only two distinct sequences and only one high resolution plane orientation per sequence, the radiologists’ assessment was limited compared to a clinical setting. Fourth, semantic properties may differ significantly between different histologies of STS. As a consequence, the prognostic value of each feature may be different depending on the histological subtype, too. This may be of particular importance as both cohorts showed a different histological distribution of histologies. This could have impaired a better performance. As a consequence, building a Semantic “histology-agnostic” prediction model may simply not be feasible. Interestingly, Radiomic models seem to extract a proportion of histology-agnostic information. Regardless, histology-specific models may be superior for patient stratification and should be investigated in the future with the expansion of our multi-institutional database, given the rarity of STS overall and the further refinement with each particular histology with over 150 different subtypes. Alternatively, if a sufficiently large cohort would be available, histology could also be added as a predictive variable itself.

The significantly improved reproducibility of the Semantic model and, to a lesser extent, the Radiomic models in the non-independent training and testing cohort (Table S9) demonstrated the impact of differing treatment, acquisition, and patient characteristics between cohorts. This validation, however, only corresponds to a TRIPOD type II validation, as differences between the cohorts become mitigated following randomized splitting of the training and testing cohort [24,50]. The usage of independent cohorts as performed in the main results corresponds to a TRIPOD type III validation, yielding a better estimate for generalizability.

In our previous study, we demonstrated the feasibility of radiomic-based prognostic risk assessment based on planning CT data, despite its inferior soft-tissue resolution [51]. In a different study, we showed the general propensity to predict OS based on T1FSGd MRI sequences using radiomics. The final model achieved a C-index of 0.68 in the independent test set [22]. In this work, we could now demonstrate that by using T2FS sequences, a similar prognostic value can be achieved without the need for contrast agent administration. However, the reported performance of our MRI models and the incremental benefit above a clinical model was lower compared to the previous results. This may be reasoned by the more stringent selection of patients based on clinical criteria (e.g., exclusion of low-grade STS and non-extremity/trunk locations), and most importantly the simultaneous presence of T1FSGd and T2FS MRI sequences leading to a 34% smaller training cohort (see the patient workflow in Figure S1).

Combing semantic and radiomic features did not improve the prediction of survival. This may be explained by the fact that radiomic features may at least partly encode tumor-specific semantic imaging features. Moreover, the total relevance of semantic features appeared to be inferior to the radiomic features when comparing model performances.

Model calibration among solely imaging-based models was suboptimal. By combing imaging models with clinical features, model calibration could be improved. As a consequence, future models should be combined with known clinical characteristics. This way, the best predictive performance and calibration can be achieved.

Improved pretherapeutic prognostic assessment of patients’ risk for systemic progression or death may help to individualize treatment regimens. Current therapy regimens of high-grade STS achieve a good LPFS by combining resection and radiotherapy. DPFS and OS, however, remain comparably low [4]. Multiple studies have analyzed the use of additional systemic therapies. For instance, multiagent chemotherapy was recently shown to be significantly associated with improved OS in a large meta-analysis of 22 studies encompassing 5044 patients [52]. However, the toxicity of these chemotherapy regimens is substantial and the total outcome remains unfavorable. Novel systemic treatment agents could be an alternative and are currently under investigation in clinical trials [53,54]. Regarding the high mutational burden of some STS entities, immunotherapeutic checkpoint inhibition may be a further option for a systemic therapy modification, which is currently being tested in the phase-II Sarc032 trial using Pembrolizumab (NCT03092323) [55]. Other molecular targeted agents, such as the MDM-2 inhibitor AMG 232 (NRG DT001 trial, NCT03217266) or trabectedin (TRASTS trial, NCT02275286), are given as a supplement to neoadjuvant RT [56,57]. Imaging-defined high-risk patients could benefit from such additional therapies, whereas low-risk patients could be spared unnecessary toxicities. The true value of such radiomic-guided therapies should be investigated in future prospective trials.

Apart from direct prognostic assessment, radiomics may be beneficial for several other tasks in STS patients. For instance, tumor characterization in terms of molecular aberrations (“radiogenomics”) or histological properties could be a potential outcome target. Multiple authors demonstrated noninvasive prediction of the important prognostic factor, “tumor grading” [58,59]. In an ongoing work, we could demonstrate promising results differentiating benign lipomas from atypical lipomatous tumors based on the murine double minutes (*MDM2*) gene amplification status. Besides, tumor response prediction may be another area of investigation by analyzing longitudinal changes in radiomic features (“delta radiomics”) parallel to RT or systemic therapies [60]. The first studies in STS and osteosarcomas demonstrated promising results [61,62].

Multiple limitations of the study should be noted that leave room for improvement of radiomic models. First, both study cohorts were collected retrospectively, constituting a reason for a potential source of bias [63]. Second, to achieve clinically homogenous patient cohorts, the patient number had to be reduced and this impaired statistical power. Third, as in many multicenter radiomic studies, the patient cohorts are prone to a substantial amount of technical heterogeneity, including a large plethora of MRI scanner types and imaging protocols. Fourth, the heterogenous histologies of STS may impair better prognostic performance for semantic, but also radiomic models. Finally, the semantic imaging features in the training and testing cohort were read by three separate readers. However, one reader read a part of each cohort and thus may have falsely increased the likeliness of overoptimistic validation between cohorts. By addressing these limitations, future research may be able to develop more effective prognostic models. As consequence, an optimal study would comprise of a large prospectively acquired patient cohort, be restricted to a predefined STS histology type, and use clearly defined MRI acquisition protocols.

## 5. Conclusions

In conclusion, we demonstrated that both MRI-based radiomic features and semantic imaging features were associated with overall survival. For radiomic models, we found that a T2FS-based radiomic model enabled prognostic assessment in addition to previous work using T1FSGd. Both models were able to achieve significant patient stratification. In comparison, the semantic model showed a decreased performance in the testing cohort. Combined semantic + radiomic models did not improve performance. Further investigation is warranted to advance towards a more personalized approach for risk-adapted tailored treatment intensification or deintensification based on imaging-based biomarkers.

## Figures and Tables

**Table 1:** Description of all semantic features extracted radiologist for the description of soft-tissue sarcomas.

Feature	Description
Anatomical region	1: chest/back, 2: neck, 3: leg, 4: gluteal/pelvis 5: arm, 6: hand, 7: foot
Localization	1: epifascial, 2: subfascial, 3: epi- and subfascial, 4: intramuscular, 5: intermuscular; 6 intra- and intermuscular
Image pattern	1: multinodular, 2: mass-like round/oval, 3: superficial spread
Borders	1: well defined /pushing type, 2: focal infiltrating, 3: diffuse infiltrating
Dominant STIR Signal intensity	1: hypointense, 2: isointense, 3: hyperintense
STIR Homogeneity	1: homogenous, 2: inhomogenous
Contrast enhancement of the tumor	1:<1/3 of the tumor, 2: 1/3–2/3, 3: >2/3
Homogeneity of Tumor contrast enhancement	1: homogeneous, 2: inhomogeneous
Tail sign	1: present, 0: absent, 2: uncertain
Vascularization	1: present, 0: absent
Necrosis	1: present, 0: absent
perilesional Edema	1: present, 0: absent
perilesional Contrast enhancement	1: present, 0: absent
Max diameter (in mm without tail)	in mm
Edema diameter (in mm)	in mm

**Table 2:** Patient demographics, outcomes, and treatment specifics.

Institution		Testing Cohort	Training Cohort	*p*-Value ^1^	*p*-Value Adjusted ^1^
**Accrual time**		2010–2016	2007–2015		
**Total Patients**		71 p	108 p		
	Primary	66 p (93%)	108 p (100%)	<0.001 *	<0.001 *
	Recurrent	5 p (7%)	0 p		
**Location**	Lower Extremity	56 p (79%)	75 p (70%)	0.36	1.0
	Upper Extremity	10 p (14%)	17 p (16%)		
	Trunk	5 p (7%)	16 p (14%)		
**Age**		m 57 (r 17–87)	m 53.7 (r 19.1–88.5)	0.16	1.0
**Gender**	female	35 p (49%)	29 (27%)	0.005 *	0.078
	male	36 p (51%)	76 (70%)		
	unknown	0 p	3 p (3%)		
**T-stage ^2^**	1	4 p (6%)	9 p (8%)	0.40	1.0
	2	30 p (42%)	32 p (30%)		
	3	23 p (32%)	41 p (38%)		
	4	14 p (20%)	26 p (24%)		
**M-stage ^2^**	0	71 p (100%)	108 p (100%)	-	-
	1	0 p (0%)	0 p (0%)		
**N-stage ^2^**	0	69 p (97%)	108 p (100%)	0.16	1.0
	1	2 p (3%)	0 p		
**Grading ^3^**	1	0 p (0%)	0 p (0%)	0.88	1.0
	2	28 p (39%)	44 p (40%)		
	3	43 p (51%)	64 p (60%)		
**AJCC-Stage ^2^**	IIA	9 p (13%)	15 (14%)	0.0025 *	0.045 *
	IIB	4 p (6%)	32 (29%)		
	III	48 p (68%)	61 (58%)		
**Margin-status**	positive	12 p (17%)	28 p (26%)	0.011	0.18
	negative	53 p (75%)	76 p (70%)		
	unknown	2 p (3%)	3 p (3%)		
	no resection	4 p (6%)	1 p (1%)		
**RT type**	post-operative	15 p (21%)	32 (29%)	<0.001 *	0.007 *
	neoadjuvant	52 p (72%)	75 p (70%)		
	definitive	4 p (6%)	1 p (1%)		
**Total RT Dose**		m 50 Gy(r 28–70 Gy)	m 50 Gy(r 38–50Gy)	<0.001 *	<0.001 *
**Chemotherapy**		3/71 p (4%)	64 p (59%)	<0.001 *	<0.001 *
**Median OS**		40.1 (r 6.0–105.5)	39.9 (r 4.2–130.4)	0.53	1.0

**Abbreviations**: *: *p*-value < 0.05, AJCC: American Joint Committee on Cancer and the International Union for Cancer Control (8th edition), m: median, p: patients, r: range, RT: radiation therapy, ^1^ Wilcoxon rank-sum test for continuous and ordinal variables, Fisher’s exact test for nominal variables, log-rank test for comparison of survival times. Corrected for multiple testing by Bonferroni correction (“*p*-value adjusted”). ^2^ Following AJCC staging system 8th edition [42]. ^3^ According to the French Federation of Cancer Centers Sarcoma Group (FNCLCC).

**Table 3:** Cox proportional hazards regression of semantic features for patients’ overall survival.

	Combined Cohort	
**Feature**	**HR (95% CI)**	***p*-Value**
Anatomic region	0.58 (0.33–1)	0.067
Localization	1.2 (0.95–1.5)	0.12
Image pattern	0.94 (0.59–1.5)	0.8
Borders	1.3 (0.86–1.9)	0.22
**Maximal diameter without tail (in mm)**	**1 (1–1)**	**0.022**
Dominant STIR signal intensity	1.3 (0.45–3.5)	0.66
STIR homogeneity	1.5 (0.74–2.9)	0.27
Tumor contrast enhancement	0.74 (0.52–1.1)	0.1
Homogeneity of Tumor contrast enhancement	1 (0.54–1.9)	0.98
Tail sign	1.5 (0.86–2.6)	0.16
Vascularization	0.95 (0.47–1.9)	0.88
**Necrosis**	**1.9 (1–3.6)**	**0.039**
Edema perilesional (in mm)	1.1 (0.6–1.9)	0.81
**Edema diameter**	**1 (1–1)**	**0.043**
Contrast enhancement perilesional	1.5 (0.85–2.6)	0.16

Univariate Cox proportional hazards regression was performed for semantic imaging features. Significant factors are written in bold. Depicted *p*-values were not corrected for multiple testing. Abbreviations: 95% CI: 95% confidence interval.

## Data Availability

The data presented in this study are available on request from the corresponding author dependent on ethics board approval. The data are not publicly available due to data protection legislation.

## Supplementary Materials

The following are available online at https://www.mdpi.com/article/10.3390/cancers13081929/s1, Figure S1: Patient workflow, Figure S2: Time-dependent AUC curves, Figure S3: Calibration curves, Table S1: Histologies of soft tissue sarcomas, Table S2: MRI acquisition parameters, Table S3: Extracted radiomic features, Table S4: R packages, Table S5: Inter-reader agreement of semantic imaging features, Table S6: Performance comparison of machine learning models, Table S7: Selected features and model coefficients, Table S8: Brier scores of developed models, Table S9: Performance in non-independent cohorts, Table S10: TRIPOD checklist
