# Supplementary material for: Prognostic Assessment in High-Grade Soft-Tissue Sarcoma Patients: A Comparison of Semantic Image Analysis and Radiomics
Source: Cancers (Basel). 2021 Apr 16;13(8):1929. doi: 10.3390/cancers13081929 (PMC8073388; doi:10.3390/cancers13081929)
Supplement: Supplementary file 1 [file cancers-13-01929-s001.zip › cancers-1147783-supplementary.pdf]

Supplemental Material

Supplemental Figures 2

Figure S1 Patients Workflow 2

Figure S2 Time dependent area under the receiver operator characteristic (ROC) curves AUCs of the developed models in the testing patient cohort 3

Figure S3 Calibration curves of the developed models in the testing patient cohort4

Figure S4 Calibration curves of the developed combined models in the testing patient cohort 5

Supplemental Tables 6

Table S1 Histologies of Soft-Tissue Sarcomas 6

Table S2 MRI acquisition parameters 7

Table S3 Extracted radiomics features 8

Table S4 R packages 11

Table S5 Inter-reader agreement of semantic imaging features. 12

Table S6 Prognostic performance of developed models depicted as concordance index with 95% confidence interval (in parenthesis). 13

Table S7 Final input variables and model coefficients of the final elastic net regression models 14

Table S9 Performance of radiomic and semantic models in randomly chosen non-independent cohorts 17

Table S10 TRIPOD Checklist: Prediction Model Development and Validation 18

Supplemental Figures

Figure S1 Patients Workflow

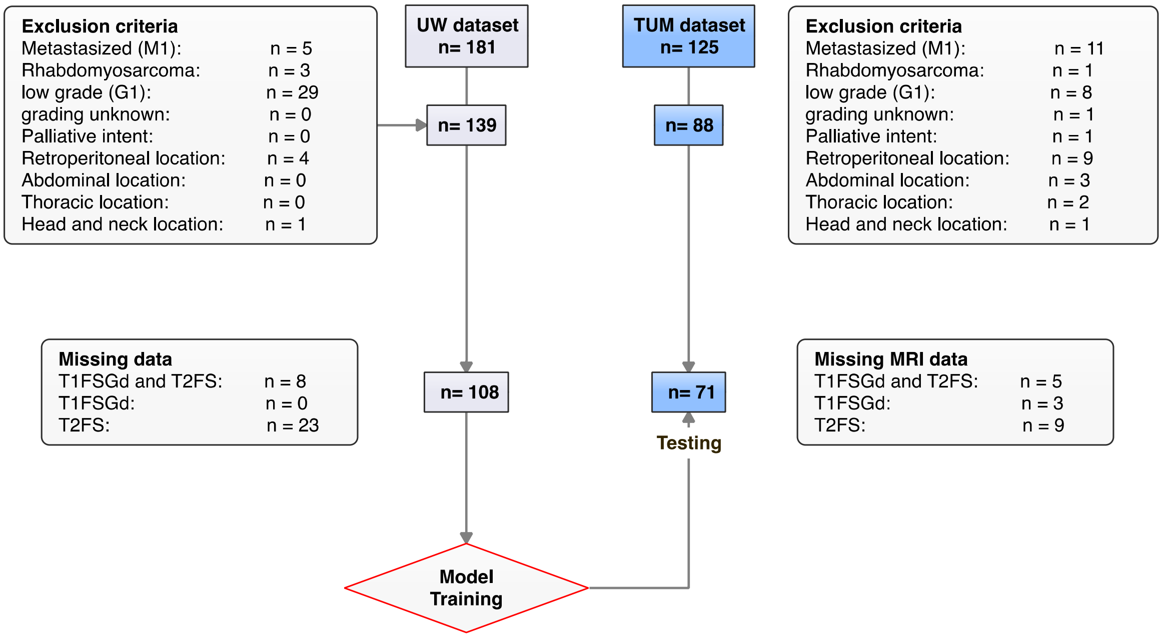

Figure S2 Time dependent area under the receiver operator characteristic (ROC) curves AUCs of the developed models in the testing patient cohort

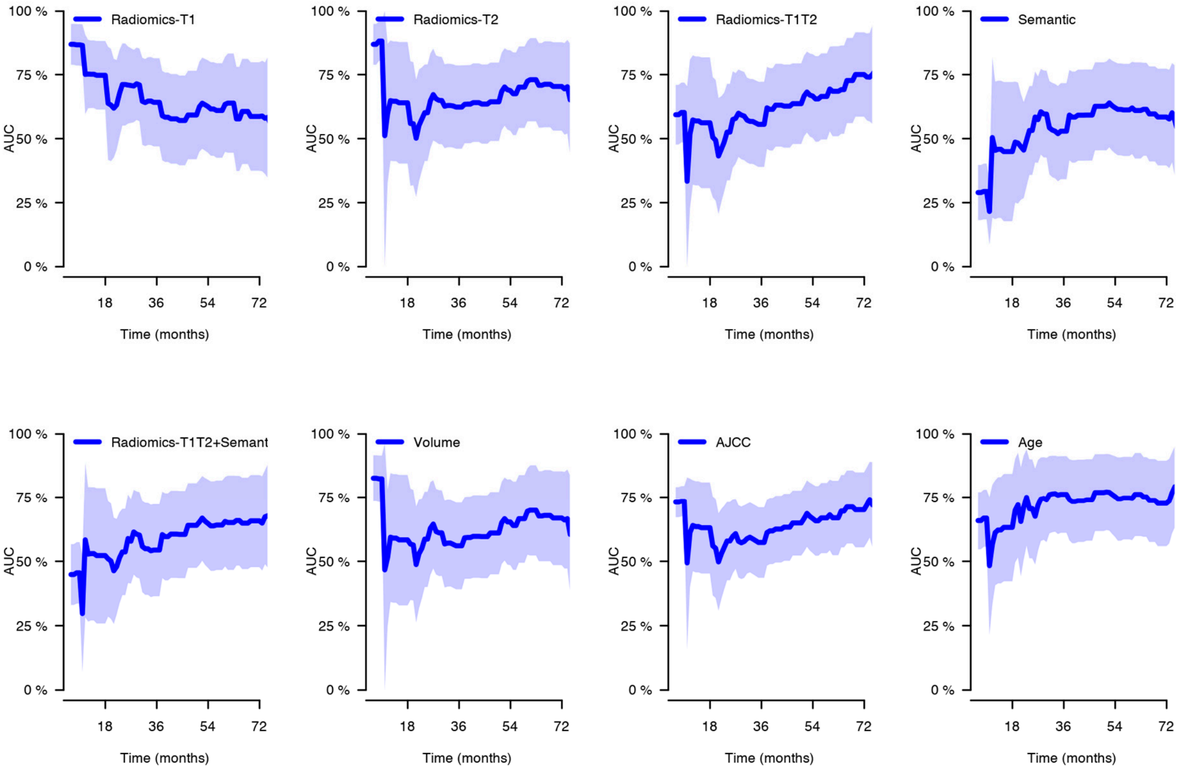

The shaded blue area represents the 95% confidence interval.

Figure S3 Calibration curves of the developed models in the testing patient cohort

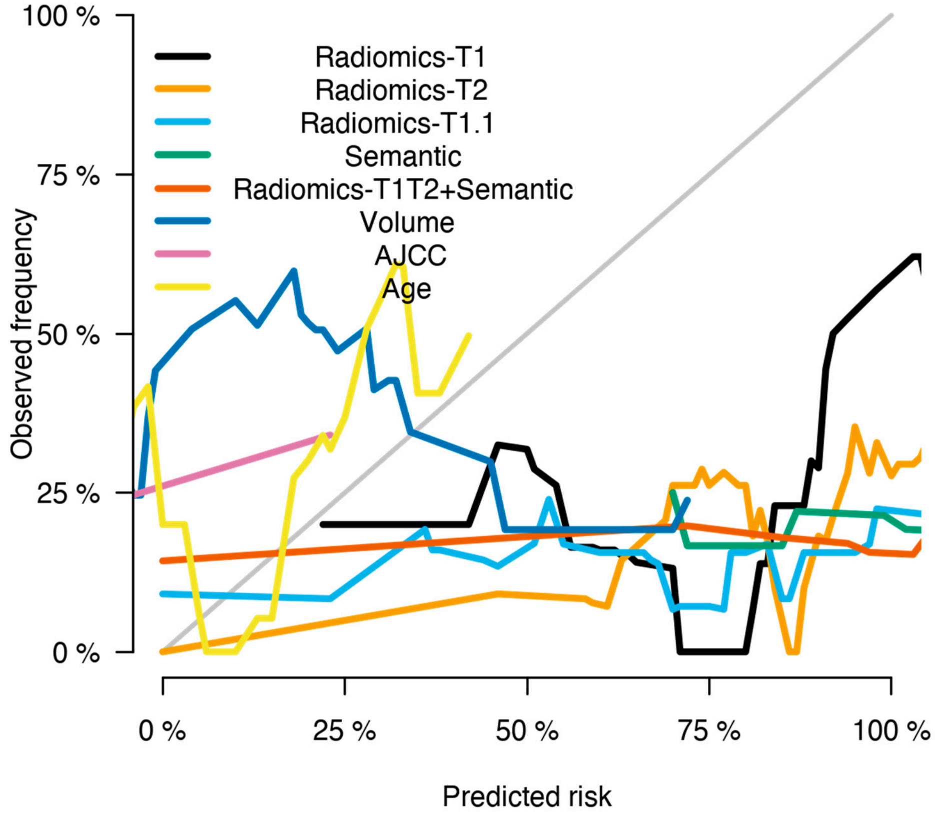

Figure S4 Calibration curves of the developed combined models in the testing patient cohort

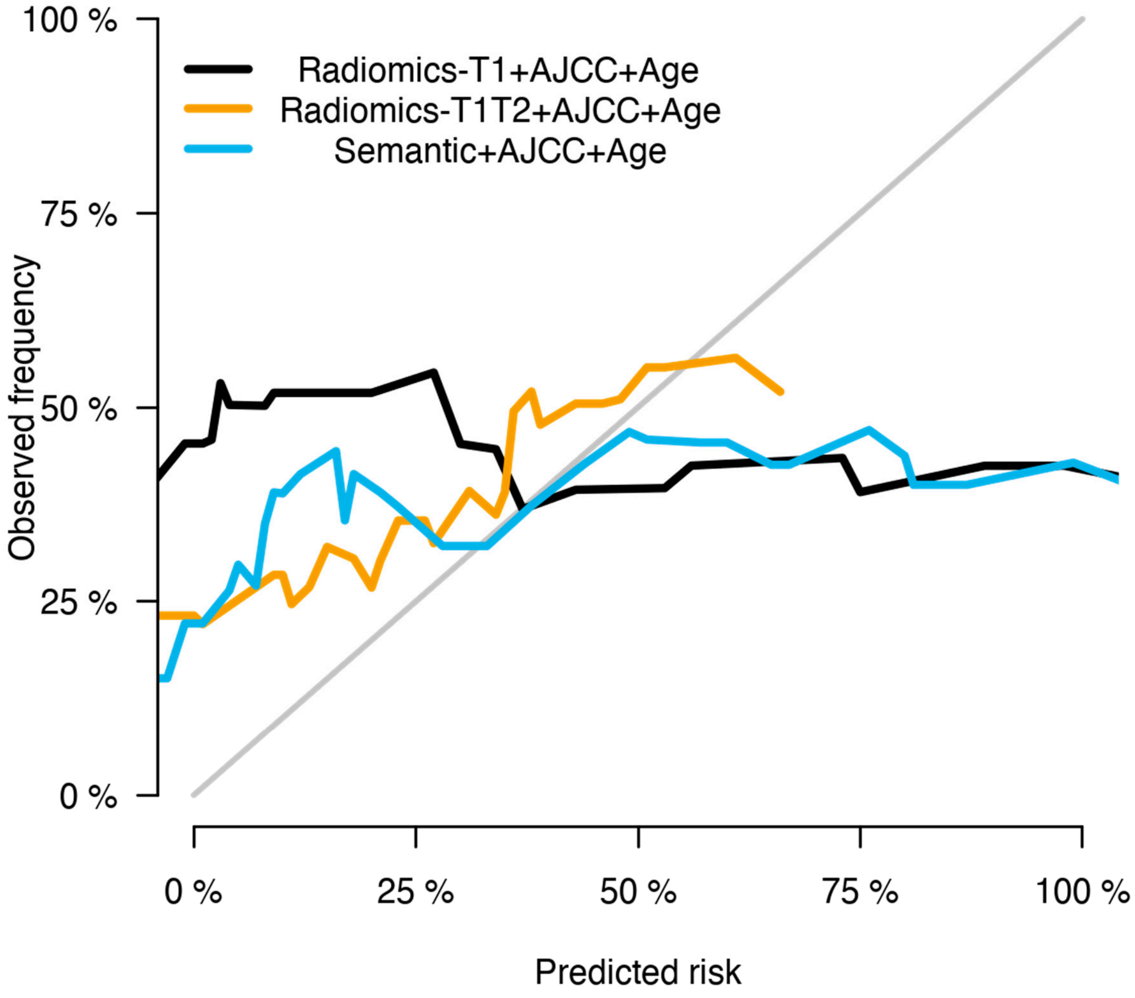

The calibration curve of Radiomics-T2+AJCC+Age is displayed in Figure 4.

Supplemental Tables

Table S1 Histologies of Soft-Tissue Sarcomas

The distribution of histologies was significantly different (p-value <0.001. Fisher's exact test). Sorted by patient number in the TUM cohort.

Histological subtype (%)	TUM (Test cohort)	UW (Training cohort)
**Pleomorphic sarcoma**	23 p (32 %)	50 p (45%)
**Myxofibrosarcoma**	13 p (18 %)	2 p (2 %)
**Synovial sarcoma**	9 p (13 %)	7 p (6 %)
**Dedifferentiated liposarcoma**	8 p (11 %)	3 p (3 %)
**Myxoid liposarcoma**	7 p (10 %)	7 p (6 %)
**Leiomyosarcoma**	3 p (4 %)	12 p (11 %)
**Liposarcoma[1]**	3 p (4 %)	2 p (2 %)
**Spindle cell sarcoma**	2 p (3 %)	10 p (10 %)
**Pleomorphic liposarcoma**	2 p (3 %)	3 p (3 %)
**Alveolar soft part sarcoma**	1 p (1 %)	0 p (0 %)
**Malignant peripheral nerve sheath tumor**	0 p (0 %)	6 p (5 %)
**Extrasekeletal myxoid Chondrosarcoma**	0 p (0 %)	2 p (2 %)
**Myofibrosarcoma**	0 p (0 %)	1 p (1 %)
**Angiosarcoma**	0 p (0 %)	1 p (1 %)
**Small round cell sarcoma**	0 p (0 %)	1 p (1 %)
**No classification**	0 p (0 %)	1 p (1 %)

[1] not further specified.

Table S2 MRI acquisition parameters
Median values are depicted. The range is noted in brackets.

	Sequence	TUM[a] (Test cohort)	UW[b] (Training cohort)
**Magnetic field strength**		1.5 T (1.5 - 3.0)	1.5 (1.0 - 3.0)
**In-Plane resolution (mm)**	**T1FSGd**	0.73 x 0.73 (0.64x0.64 -0.81x0.81)	0.56 x 0.56 (0.20 x 0.20 - 1.56 x 1.56)
**Slice thickness (mm)**		5 (4 - 5)	6.5 (1.0 - 11.0)
**Matrix**		320 x 320 (176 x 176 - 1152 x 1152)	512 x 512 (256 x 190 – 1200 x 1200)
**TR (ms)**		685 (500 - 1311)	645 (150 – 1533)
**TE (ms)**		12 (4 - 20)	14 (9 - 29)
**Plane Orientation**		axial: 70 p (99%) sagittal: 1 p (1%) coronal: 0 p (0%)	axial: 106 p (98 %) sagittal: 1 p (1 %) coronal: 1 p (1 %)
**In-Plane resolution (mm)**	**T2FS**	0.73 x 0.73 (0.64x0.64 -0.81x0.81)	0.625 x 0.625 (0.17 x 0.17 - 1.72 x 1.72)
**Slice thickness (mm)**		5 (4 - 5)	6.5 (3.3 - 11.6)
**Matrix**		448 x 448 (222 x 222 - 1200 x 1200)	432 x 432 (160 x 224 - 880 x 880)
**TR (ms)**		7330 (2021 - 18516)	3925 (370 -13000)
**TE (ms)**		60 (18 - 133)	64.5 (12-738)
**Plane Orientation**		axial: 7 p (10%) sagittal: 5 p (7%) coronal: 59 p (83%)	axial: 103 p (95 %) sagittal: 3 p (3 %) coronal: 2 p (2 %)

Abbreviation: p: patients, T1FSGd: contrast-enhanced T1-weighted fat saturated, T2FS: T2-weighted fat saturated, TE: echo time, TR: repetition time,
[a]MRI Scanner: GE (Chicago, USA): Signa; Philips (Amsterdam, Netherlands): Achieva, Ingenia; Siemens (Munich, Germany): Verio, Avanto, Symphony,
[b]MRI Scanner: GE (Chicago, USA): Discovery MR750, Signa, Optima 450; Hitachi (Tokyo, Japan): Oasis; Philips (Amsterdam, Netherlands): Achieva, Gyroscan NT, Ingenia, Intera; Siemens (Munich, Germany): Avanto, Area, Espree, Harmony, Symphony, TrioTim; Toshiba (Tokyo, Japan): Titan.

Extracted radiomic features

All over 105 features per sequences were extract from the original image or the labelmap (shape features) yielding a total of 210 features per patients. The pyradiomics package (version 2.2) implemented in python was used to calculate all features. The features were calculated following the Imaging Biomarker Standardization Initiative (IBSI) [1]. Table S3 depicts all extracted features. Please see the pyradiomics documentation (http://pyradiomics.readthedocs.io/en/latest/features.html) for further details such as formulae.

Table S3 Extracted radiomics features

	**Shape Features**
1.)	Volume
2.)	Surface Area
3.)	Surface Volume Area
4.)	Sphericity
5.)	Spherical Disproportion
6.)	Maximum 3D Diameter
7.)	Maximum 2D Diameter Slice
8.)	Maximum 2D Diameter Column
9.)	Maximum 2D Diameter Row
10.)	Major Axis
11.)	Minor Axis
12.)	Least Axis
13.)	Elongation
14.)	Flatness
	**First Order Features**
1.)	Energy
2.)	Intensity Histogram Entropy
3.)	Minimum
4.)	10th Percentile
5.)	90th Percentile
6.)	Maximum
7.)	Mean
8.)	Median
9.)	Interquartile Range
10.)	Range
11.)	Mean Absolute Deviation (MAD)
12.)	Robust Mean Absolute Deviation (rMAD)
13.)	Root Mean Squared (RMS)
14.)	Skewness
15.)	Excess Kurtosis
16.)	Variance
17.)	Intensity Histogram Uniformity
	**Gray Level Co-occurrence Matrix (GLCM) Features**
1.)	Autocorrelation

2.)	Joint Average
3.)	Cluster Prominence
4.)	Cluster Shade
5.)	Cluster Tendency
6.)	Contrast
7.)	Correlation
8.)	Difference Average
9.)	Difference Entropy
10.)	Difference Variance
11.)	Joint Energy (IBSI: Angular Second Moment)
12.)	Joint Entropy
13.)	Informal Measure of Correlation (IMC) 1
14.)	Informal Measure of Correlation (IMC) 2
15.)	Inverse Difference Moment (IDM)
16.)	Inverse Difference Moment Normalized (IDMN)
17.)	Inverse Difference (ID)
18.)	Inverse Difference Normalized (IDN)
19.)	Inverse Variance
20.)	Maximum Probability (IBSI: Joint maximum)
21.)	Sum Entropy
22.)	Sum of Squares (IBSI: Sum of Squares)
23.)	Maximal Correlation Coefficient (MCC)
	**Gray Level Size Zone Matrix (GLSZM) Features**
1.)	Small Area Emphasis (SAE)
2.)	Large Area Emphasis (LAE)
3.)	Gray Level Non-Uniformity (GLN)
4.)	Gray Level Non-Uniformity Normalized (GLNN)
5.)	Size-Zone Non-Uniformity (SZN)
6.)	Size-Zone Non-Uniformity Normalized (SZNN)
7.)	Zone Percentage (ZP)
8.)	Gray Level Variance (GLV)
9.)	Zone Variance (ZV)
10.)	Zone Entropy (ZE)
11.)	Low Gray Level Zone Emphasis (LGLZE)
12.)	High Gray Level Zone Emphasis (HGLZE)
13.)	Small Area Low Gray Level Emphasis (SALGLE)
14.)	Small Area High Gray Level Emphasis (SAHGLE)
15.)	Large Area Low Gray Level Emphasis (LALGLE)
16.)	Large Area High Gray Level Emphasis (LAHGLE)
	**Gray Level Run Length Matrix (GLRLM) Features**
1.)	Short Run Emphasis (SRE)
2.)	Long Run Emphasis (LRE)
3.)	Gray Level Non-Uniformity (GLN)
4.)	Gray Level Non-Uniformity Normalized (GLNN)
5.)	Run Length Non-Uniformity (RLN)
6.)	Run Length Non-Uniformity Normalized (RLNN)
7.)	Run Percentage (RP)

8.)	Gray Level Variance (GLV)
9.)	Run Variance (RV)
10.)	Run Entropy (RE)
11.)	Low Gray Level Run Emphasis (LGLRE)
12.)	High Gray Level Run Emphasis (HGLRE)
13.)	Short Run Low Gray Level Emphasis (SRLGLE)
14.)	Short Run High Gray Level Emphasis (SRHGLE)
15.)	Long Run Low Gray Level Emphasis (LRLGLE)
16.)	Long Run High Gray Level Emphasis (LRHGLE)
	**Neighbouring Gray Tone Difference Matrix (NGTDM) Features**
1.)	Coarseness
2.)	Contrast
3.)	Busyness
4.)	Complexity
5.)	Strength
	**Gray Level Dependence Matrix (GLDM) Features**
1.)	Small Dependence Emphasis (SDE)
2.)	Large Dependence Emphasis (LDE)
3.)	Gray Level Non-Uniformity (GLN)
4.)	Dependence Non-Uniformity (DN)
5.)	Dependence Non-Uniformity Normalized (DNN)
6.)	Gray Level Variance (GLV)
7.)	Dependence Variance (DV)
8.)	Dependence Entropy (DE)
9.)	Low Gray Level Emphasis (LGLE)
10.)	High Gray Level Emphasis (HGLE)
11.)	Small Dependence Low Gray Level Emphasis (SDLGLE)
12.)	Small Dependence High Gray Level Emphasis (SDHGLE)
13.)	Large Dependence Low Gray Level Emphasis (LDLGLE)
14.)	Large Dependence High Gray Level Emphasis (LDHGLE)

Table S4 R packages

Package	Purpose	Version
Irr	inter-rater agreement	0.84.1
ggkm	kaplan meier survival curves	1.0
ggplot2	figures	3.1.0
glmnet	least absolute shrinkage and selection operator	2.0-16
glmnutils	elastic net regression	1.1.2
icc	intra-class coefficient	2.3.0
boruta	boruta feature reduction	7.0.0
dummies	one hot encoding	1.5.6
mlr3	nested cross validation	0.5.0
mlr3proba	survival analysis	0.1.4
mlrtuning	tuning for mlr2	0.3.0
boot	bootstrapping	1.3-20
survival	survival models	2.43-3
Survminer	cox mode plotting	0.4.8
riskregression	time-dependent AUC, calibration curves	2020.02.05

Table S5 Inter-reader agreement of semantic imaging features.
10 soft-tissue sarcoma patients were randomly selected from the testing cohort. Three blinded radiologist performed semantic feature extraction. Display of the Fleiss Kappa statistic for nominal and ordinal features and the intra-class coefficient (ICC) for continual measures.

Feature	Fleiss Kappa
Anatomical region	1.000
Necrosis	1.000
Boarders	0.733
Homogeneity of Tumor contrast enhancement	0.683
Tumor contrast enhancement	0.641
Dominant STIR Signalintensity	0.535
Edema-perilesional	0.524
Localization	0.468
STIR Homogeneity	0.444
Image pattern	0.400
Tail sign	0.334
Contrast enhancement perilesional	0.047
Vascularization	-0.035
	**ICC**
Edema diameter (in mm)	0.846
Max diameter (in mm wo/ tail)	0.833

Table S6 Prognostic performance of developed models depicted as concordance index with 95% confidence interval (in parenthesis).

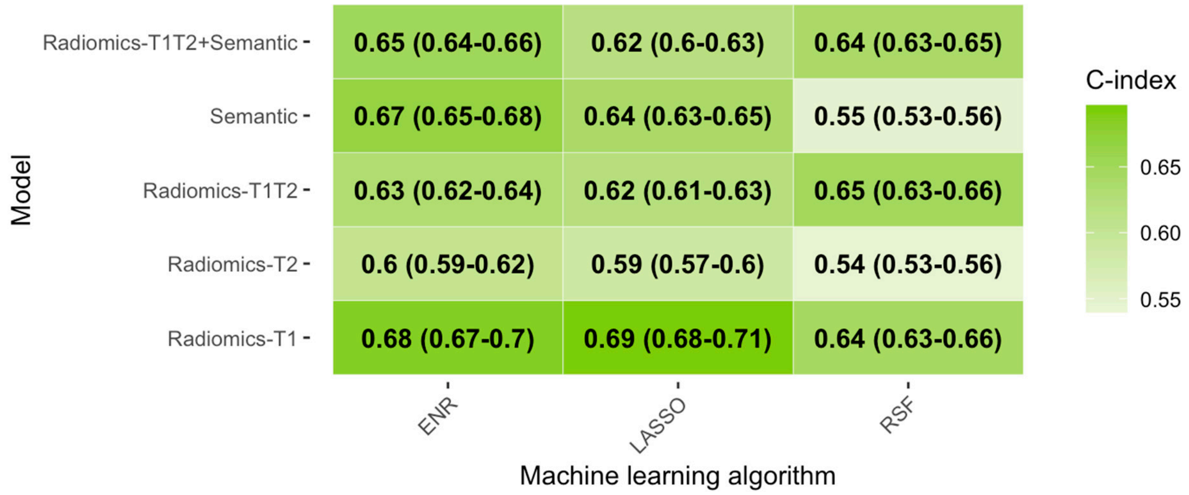

Abbreviations: ENR: elastic net regression. LASSO: least absolute shrinkage and selection operator. RSF: random survival forest.

Table S7 Final input variables and model coefficients of the final elastic net regression models

Displayed beta coefficients are based on the standardized features.

Feature	B coefficient
**Radiomics-T1**	
original_firstorder_Mean	-0.4066929
original_shape_SurfaceArea	0.2425698
original_gldm_SmallDependenceHighGrayLevelEmphasis	0
original_gldm_LargeDependenceLowGrayLevelEmphasis	0
original_gldm_LowGrayLevelEmphasis	0
original_shape_Maximum2DDiameterSlice	0
original_shape_Elongation	0
original_shape_Flatness	0
original_glszm_HighGrayLevelZoneEmphasis	0
original_gldm_HighGrayLevelEmphasis	0
original_glszm_GrayLevelVariance	0
original_gldm_SmallDependenceLowGrayLevelEmphasis	0
original_gldm_GrayLevelNonUniformity	0
**Radiomics-T2**	
original_glszm_SizeZoneNonUniformity	0.140311012
original_gldm_LargeDependenceLowGrayLevelEmphasis	0.113575289
original_glszm_LargeAreaLowGrayLevelEmphasis	0.100207348
original_gldm_GrayLevelNonUniformity	0.092784326
original_glszm_SmallAreaEmphasis	-0.07906781
original_gldm_SmallDependenceHighGrayLevelEmphasis	-0.03391476
original_shape_Flatness	-0.03074633
original_gldm_SmallDependenceEmphasis	-0.01761524
original_glszm_ZonePercentage	-0.0077572
original_ngtdm_Busyness	0
**Radiomics-T1T2**	
original_glszm_SizeZoneNonUniformity_T2FS	0.34867721
original_gldm_LargeDependenceLowGrayLevelEmphasis_T2FS	0.31184005
original_glcm_MCC_T2FS	0.22502526
original_gldm_LowGrayLevelEmphasis_T1FSGd	0.21135891
original_shape_Flatness_T1FSGd	-0.06965916
original_firstorder_Kurtosis_T1FSGd	-0.04694111
original_gldm_LargeDependenceLowGrayLevelEmphasis_T1FSGd	0
original_shape_Flatness_T2FS	0
original_shape_Elongation_T1FSGd	0
original_ngtdm_Busyness_T2FS	0
original_gldm_SmallDependenceLowGrayLevelEmphasis_T1FSGd	0
**Semantic**	

Localization 1: epifascial	-0.82732997
Necrosis	0.462067102
Contrast enhancement perilesional	0.445673686
Localization 4: intramuscular	0.305219026
Tail sign	0.206750047
Anatomical region 3: leg	-0.19335878
Tumor contrast enhancement (<1/3;1/3-2/3;>2/3)	-0.15002045
Max diameter (in mm wo/ tail)	0.002050004
Edema diameter (in mm)	0.000549519
**Radiomics-T1T1+Semantic**	
Localization_T1FSGd	-0.4307884
Contrast enhancement perilesional	0.295373492
Necrosis	0.205045814
Localization 4: intramuscular	0.186908909
original_gldm_LargeDependenceLowGrayLevelEmphasis_T2FS	0.15237439
Tail sign	0.150061231
original_glcm_MCC_T2FS	0.125961179
original_gldm_LowGrayLevelEmphasis_T1FSGd	0.113698266
original_glszm_SizeZoneNonUniformity_T2FS	0.100942757
Anatomical region 3: leg	-0.0849289
original_firstorder_Kurtosis_T1FSGd	-0.05369461
Tumor contrast enhancement	-0.05337103
original_shape_Flatness_T1FSGd	-0.02081777
original_shape_Flatness_T2FS	-0.01964066
original_ngtdm_Busyness_T2FS	0.010474745
original_shape_Elongation_T1FSGd	-0.00413689
original_gldm_LargeDependenceLowGrayLevelEmphasis_T1FSGd	0.003302509
Edema diameter (in mm)	0.000346507
Max diameter (in mm wo/ tail)	0.000326452
original_gldm_SmallDependenceLowGrayLevelEmphasis_T1FSGd	0

Table S8 Brier scores of developed models

Model	Brier score
Radiomics-T1	88
Radiomics-T2	71
Radiomics-T1T2	115
Semantic	702
Radiomics-T1T2+Semantic	371
Volume	3646
AJCC	30
Age	24
Radiomics-T1+AJCC+Age	53
Radiomics-T2+AJCC+Age	41
Radiomics-T1T2+AJCC+Age	37
Semantic+AJCC+Age	56

Table S9 Performance of radiomic and semantic models in randomly chosen non-independent cohorts

Model	Training	Testing
Radiomics-T1	0.63 (0.62-0.64)	0.63 (0.49-0.76)
Radiomics-T2	0.63 (0.62-0.64)	0.63 (0.49-0.76)
Radiomics-T1T2	0.56 (0.55-0.58)	0.65 (0.51-0.77)
Semantic	0.64 (0.63-0.66)	0.63 (0.49-0.78)
Radiomics-T1T2+Semantic	0.61 (0.6-0.63)	0.64 (0.51-0.78)

Patient cohort

C-index: 0.58, 0.60, 0.62, 0.64

Table S10 TRIPOD Checklist: Prediction Model Development and Validation

**Section/Topic**			**Checklist Item**	**Page**
**Title and abstract**				
Title	1	D;V	Identify the study as developing and/or validating a multivariable prediction model, the target population, and the outcome to be predicted.	1
Abstract	2	D;V	Provide a summary of objectives, study design, setting, participants, sample size, predictors, outcome, statistical analysis, results, and conclusions.	1
**Introduction**				
Background and objectives	3a	D;V	Explain the medical context (including whether diagnostic or prognostic) and rationale for developing or validating the multivariable prediction model, including references to existing models.	3
	3b	D;V	Specify the objectives, including whether the study describes the development or validation of the model or both.	3
**Methods**				
Source of data	4a	D;V	Describe the study design or source of data (e.g., randomized trial, cohort, or registry data), separately for the development and validation data sets, if applicable.	3
	4b	D;V	Specify the key study dates, including start of accrual; end of accrual; and, if applicable, end of follow-up.	7
Participants	5a	D;V	Specify key elements of the study setting (e.g., primary care, secondary care, general population) including number and location of centres.	3
	5b	D;V	Describe eligibility criteria for participants.	3
	5c	D;V	Give details of treatments received, if relevant.	7
Outcome	6a	D;V	Clearly define the outcome that is predicted by the prediction model, including how and when assessed.	3,5
	6b	D;V	Report any actions to blind assessment of the outcome to be predicted.	4
Predictors	7a	D;V	Clearly define all predictors used in developing or validating the multivariable prediction model, including how and when they were measured.	6
	7b	D;V	Report any actions to blind assessment of predictors for the outcome and other predictors.	4
Sample size	8	D;V	Explain how the study size was arrived at.	3, Fig S1
Missing data	9	D;V	Describe how missing data were handled (e.g., complete-case analysis, single imputation, multiple imputation) with details of any imputation method.	3
Statistical analysis methods	10a	D	Describe how predictors were handled in the analyses.	4-5
	10b	D	Specify type of model, all model-building procedures (including any predictor selection), and method for internal validation.	5-6
	10c	V	For validation, describe how the predictions were calculated.	6
	10d	D;V	Specify all measures used to assess model performance and, if relevant, to compare multiple models.	6,7
	10e	V	Describe any model updating (e.g., recalibration) arising from the validation, if done.	-
Risk groups	11	D;V	Provide details on how risk groups were created, if done.	7
Development vs. validation	12	V	For validation, identify any differences from the development data in setting, eligibility criteria, outcome, and predictors.	6
**Results**				
Participants	13a	D;V	Describe the flow of participants through the study, including the number of participants with and without the outcome and, if applicable, a summary of the follow-up time. A diagram may be helpful.	Fig S1
	13b	D;V	Describe the characteristics of the participants (basic demographics, clinical features, available predictors), including the number of participants with missing data for predictors and outcome.	Table 2, p 7
	13c	V	For validation, show a comparison with the development data of the distribution of important variables (demographics, predictors and outcome).	Table 2, p 7
Model development	14a	D	Specify the number of participants and outcome events in each analysis.	Table 2, p 7
	14b	D	If done, report the unadjusted association between each candidate predictor and outcome.	9, 12
Model specification	15a	D	Present the full prediction model to allow predictions for individuals (i.e., all regression coefficients, and model intercept or baseline survival at a given time point).	T S7 coeff.
	15b	D	Explain how to the use the prediction model.	-
Model performance	16	D;V	Report performance measures (with CIs) for the prediction model.	9,11
Model-updating	17	V	If done, report the results from any model updating (i.e., model specification, model performance).	-
**Discussion**				
Limitations	18	D;V	Discuss any limitations of the study (such as nonrepresentative sample, few events per predictor, missing data).	14
Interpretation	19a	V	For validation, discuss the results with reference to performance in the development data, and any other validation data.	12,13
	19b	D;V	Give an overall interpretation of the results, considering objectives, limitations, results from similar studies, and other relevant evidence.	12
Implications	20	D;V	Discuss the potential clinical use of the model and implications for future research.	14
**Other information**				
Supplementary information	21	D;V	Provide information about the availability of supplementary resources, such as study protocol, Web calculator, and data sets.	14
Funding	22	D;V	Give the source of funding and the role of the funders for the present study.	14

References

[1] Zwanenburg A. Vallières M. Abdalah MA. Aerts HJWL. Andrearczyk V. Apte A. et al. The Image Biomarker Standardization Initiative: Standardized Quantitative Radiomics for High-Throughput Image-based Phenotyping. Radiology 2020:191145. doi:10.1148/radiol.2020191145.
